# Osteoclasts in Multiple Myeloma Are Derived from Gr-1+CD11b+Myeloid-Derived Suppressor Cells

**DOI:** 10.1371/journal.pone.0048871

**Published:** 2012-11-16

**Authors:** Junling Zhuang, Jianghong Zhang, Seint T. Lwin, James R. Edwards, Claire M. Edwards, Gregory R. Mundy, Xiangli Yang

**Affiliations:** 1 Department of Medicine & Pharmacology, Vanderbilt University Medical Center, Nashville, Tennessee, United States of America; 2 Department of Cancer Biology, Vanderbilt University School of Medicine, Nashville, Tennessee, United States of America; 3 Department of Hematology, Peking Union Medical College Hospital, Beijing, China; University of Southern California, United States of America

## Abstract

Osteoclasts play a key role in the development of cancer-associated osteolytic lesions. The number and activity of osteoclasts are often enhanced by tumors. However, the origin of osteoclasts is unknown. Myeloid-derived suppressor cells (MDSCs) are one of the pre-metastatic niche components that are induced to expand by tumor cells. Here we show that the MDSCs can differentiate into mature and functional osteoclasts in vitro and in vivo. Inoculation of 5TGM1-GFP myeloma cells into C57BL6/KaLwRij mice led to a significant expansion of MDSCs in blood, spleen, and bone marrow over time. When grown in osteoclastogenic media in vitro, MDSCs from tumor-challenged mice displayed 14 times greater potential to differentiate into mature and functional osteoclasts than those from non-tumor controls. Importantly, MDSCs from tumor-challenged *LacZ* transgenic mice differentiated into LacZ+osteoclasts in vivo. Furthermore, a significant increase in tumor burden and bone loss accompanied by increased number of osteoclasts was observed in mice co-inoculated with tumor-challenged MDSCs and 5TGM1 cells compared to the control animals received 5TGM1 cells alone. Finally, treatment of MDSCs from myeloma-challenged mice with Zoledronic acid (ZA), a potent inhibitor of bone resorption, inhibited the number of osteoclasts formed in MDSC cultures and the expansion of MDSCs and bone lesions in mice. Collectively, these data provide in vitro and in vivo evidence that tumor-induced MDSCs exacerbate cancer-associated bone destruction by directly serving as osteoclast precursors.

## Introduction

It has long been recognized that dissemination of tumor cells to specific organ sites is a prerequisite for metastasis [1], [2]. However, the changes occurring in metastatic sites that support metastatic cancer cell growth have been largely under investigated. Recent observations have shown that primary tumor growth can induce changes in non-malignant cells at distant sites, forming what is known as a “pre-metastatic niche” [3], [4], to which tumor cells can home and develop. Although data related to the establishment of a pre-metastatic niche in bone are scarce, studies have shown that osteoclasts, which are derived from myeloid hematopoietic progenitor cells and responsible for bone resorption of bone matrix during normal bone remodeling [5], are critical for the engraftment of tumor cells within the bone marrow [2].

Myeloid-derived suppressor cells (MDSCs) are a heterogeneous population of cells that are defined by their myeloid origin, immature state, and ability to potently suppress T lymphocyte responses. In mice, Gr-1 or Ly-6G/Ly-6C (a granulocyte marker) and CD11b or Mac-1 (macrophage lineage marker) are two widely used surface markers for MDSCs. In healthy mice, the majority of MDSCs reside in the bone marrow and only a small proportion (<4%) of these cells can be found in the blood and spleen. They regulate immune responses and tissue repair in healthy individuals and the population rapidly expands during inflammation, infection and cancer [6], [7]. Recent in vivo evidence demonstrated that Gr-1+/CD11b+cells are important cellular components that promote tumor development and metastasis by forming tumor endothelium, releasing MMP-9 or suppressing T cell or NK cell function [8]–[11].

Multiple myeloma (MM) is a fatal cancer that develops within the bone marrow by uncontrolled proliferation of malignant plasma cells and patients suffer from bone loss caused by excessive osteoclast activity [12], [13]. When cancer cells grow in bone in MM or cancer-bone metastasized condition, they initiate a “vicious cycle” by secreting growth factors and cytokines to induce osteoclast differentiation and excessive bone resorption that in turn leads to an increase the production of growth factors, such as transforming growth factor beta (TGFβ), to stimulate tumor growth in bone [14], [15]. Therefore, osteoclasts are critical for the establishment of vicious cycle. They control not only bone destruction associated with cancer, but also the aggressive behavior of tumor cells [16]. The osteoclasts are a tissue-specific multinucleated macrophages differentiated from monocyte/macrophage precursor cells [17]. Murine CD11b+cells from bone marrow or spleens can form osteoclasts whereas human circulating CD14+monocytes can be induced to differentiate into osteoclasts [18]. However, the sources responsible for the increased number and activity of osteoclasts in disease conditions remain to be unexplored.

Given the myeloid origin of osteoclasts and the fact that cancer cells stimulate the expansion of Gr-1+/CD11b+or MDSCs, we hypothesized that tumor-induced MDSCs can give rise to osteoclasts. Using 5TGM1 murine myeloma cells and several mouse models, we investigated whether MM cells could increase the Gr-1+/CD11b+cell population in mice and further compared the capacities of naïve and tumor-associated MDSCs to differentiate into functional osteoclasts. Furthermore, we explored whether tumor-associated MDSCs promoted myeloma burden and bone destruction. Finally, the effect of Zoledronic acid, a potent inhibitor of osteoclast differentiation and function, on myeloma-associated Gr-1+/CD11b+expansion and osteoclast differentiation was also examined.

## Materials and Methods

### Cell Line and Animal Models

5TGM1-GFP myeloma cell line was cultured as described previously [19]. Animal studies were approved by the Institute of Animal Care and Use Committees at Vanderbilt University and conducted in accordance with the National Institutes of Health (NIH) Guide for the Care and Use of Laboratory Animals. Eight to 10-week-old female C57BL6/KaLwRij mice (Harlan Netherlands, Horst, The Netherlands, [20]) were used as 5TGM1 myeloma model. We also used *Rag2* ^−/−^ immunodeficient mice in our study since 5TGM1 cells induced myeloma in *Rag2* ^−/−^ mice similarly to that in C57BL6/KaLwRij mice [21]. *Lac*Z transgenic mice in the *Rag2* ^−/−^ background were generated by crossing the C57BL6.129S7-*Gt(ROSA)26Sor*/J *lac*Z heterozygous mice (The Jackson Laboratory, Bar Harbor, ME) and C57BL6 *Rag2* ^−/−^ homozygous mice (the TACONIC, Hudson, NY). Genotyping was performed by PCR using DNA isolated from mouse tails as template and primer pairs listed as the following: 5′-GGG AGG ACA CTC ACT TGC CAG TA (Rag A) and 5′-AGT CAG GAG TCT CCA TCT CAC TGA (Rag B) for *Rag2* wild type allele; 5′ CGG CCG GAG AAC CTG CGT GCA A (Neo A) and Rag B for *Rag2* mutant allele; 5′-GGC TTA AAG GCT AAC CTG ATG TG (Ros A) and 5′-GGA GCG GGA GAA ATG GAT ATG (Ros B) for ROSA 26 *lac*Z wild type allele; and 5′-AAT CCA TCT TGT TCA ATG GCC GAT C (LacZ A) and 5′-CCG GAT TGA TGG TAG TGG TC (LacZ B) for transgenic allele.

### Myeloma Tumor Induction

Disseminated myeloma was induced by intravenous tail vein inoculation of 1×10^6^ 5TGM1-GFP cells in 100 µl phosphate-buffered saline (PBS) into C57B6/KaLwRij mice. Non-tumor bearing control (naïve) mice were similarly inoculated with PBS vehicle. Following tumor cell inoculation, control or tumor-bearing mice were sacrificed on day 7, -14, -21 and -28 post implantation. GFP+tumor cells were counted and level of monoclonal mouse IgG2bκ paraprotein concentrations were measured as described previously [19].

For co-transplantation experiments, C57 BL6/KaLwRijHsd or *Rag2* ^−/−^ mice were inoculated with 5TGM1-GFP myeloma cells (1×10^6^) and Gr-1+/CD11b+cells (1×10^6^) isolated from non-tumor (control) or myeloma-bearing mice. The experimental end-point was determined when the first mouse developed paralysis of lower limb. All mice were subsequently sacrificed.

### Flow Cytometry

Cells were isolated from bone marrow and spleen of naïve control and 5TGM1 myeloma-bearing mice and filtered through a 70-µm cell strainer (BD Falcon, Bedford, MA). Cells were labeled with anti-Gr-1-PE (Miltenyi Biotec, Auburn, CA) and anti-CD11b-cy5 (BD Biosciences, San Jose, CA) then analyzed for Gr-1+/CD11b+cells in non-GFP cells (non-tumor cells). GFP fluorescence was detected to evaluate tumor burden by using a 3 laser BD LSRII (Becton Dickinson, San Jose, CA).

For cell sorting, because all Gr-1+cells are CD11b+, we used anti-Gr-1-PE monoclonal antibody (Miltenyi Biotec, Auburn, CA) and anti-PE MACS microbeads (Miltenyi Biotec, Auburn, CA) to sort Gr-1+/CD11b+cells. Briefly, 10 µl of anti-Gr-1-PE were added to 10^7^ splenocytes in 100 µl of magnetic-activated cell sorting (MACS) buffer (1× PBS supplemented with 2 mM EDTA and 0.5% bovine serum albumin) at 4°C in the dark for 10 min. Splenocytes were washed twice with MACS buffer. Ten µl of anti-PE MACS microbeads were added to 10^7^ splenocytes in 100 µl of MACS buffer at 4°C in dark for 15 min. Cells were washed twice with MACS buffer and passed through a column in a magnetic field (Miltenyi Biotec, Auburn, CA). The Gr-1+cells in the column were collected by removing the magnetic field and flushed with PBS.

### In vitro Osteoclast Differentiation and Resorption Assays

One hundred µl PBS containing 1×10^5^ cells (for differentiation assays) or 200 µl containing 2×10^5^ cells (for resorption assays) of Gr-1+/CD11b+cell suspension in α-minimum essential medium (αMEM) supplemented with 10% fetal bovine serum (FBS), 2 mmol/l L-glutamine, 100 U/ml penicillin-streptomycin were plated in a 96-well tissue culture plate containing round glass cover slips (Electron Microscopy Sciences, Hatfield, PA) or dentine slices (IDS Ltd, Boldon, UK). After 3 hours’ incubation, cover slips and dentine slices were washed in α-MEM to remove non-adherent cells and then placed in a 24-well tissue culture plate containing 1 ml of α-MEM/FBS supplemented with 25 ng/ml recombinant murine macrophage-colony stimulating factor (M-CSF) and 50 ng/ml soluble receptor activator of NF-κB ligand (RANKL) (PeproTech Inc, Rocky Hill, NJ). Cultures were incubated for up to 14 days and 28 days for differentiation and resorption assays, respectively. The culture medium was replenished every 2–3 days. Tartrate-resistant acid phosphatase (TRAP) kit (Sigma-Aldrich, St Louis, MO) was used for staining of TRAP, a cell surface marker for osteoclasts, according to the manufacture’s procedure. For immunocytochemistry, monoclonal primary antibodies, anti-Gr-1, anti-CD11b, and anti-VNR α_v_ chain (CD51) and anti-rat Ig HRP detection kit (BD biosciences, San Jose, CA) were used. Both primary and secondary antibodies were diluted to 1∶50. Polyclonal anti-CRLR (rabbit) against calcitonin receptor-like receptor (1∶50) and goat anti-rabbit HRP (1∶100, Santa Cruz Biotechnology, Inc. Santa Cruz, CA) were used to detect calcitonin receptor-like receptor. DAB peroxidase substrate kit (Vector Laboratories, Inc. Burlingame, CA) was used for chromogen development.

The active resorption of the multinucleated TRAP+cells was accessed by quantifying resorbed areas on the Gr-1+/CD11b+cells cultured on dentine discs for 28 days. Dentine slices were rinsed in PBS and placed in 1.0 M ammonium hydroxide for 24 hours and sonicated for 2 minutes to remove cells. The slices were then washed in distilled water, stained with 0.5% toluidine blue in 0.5% aqueous boric acid, and examined under light microscopy. Lacunar area/total area was analyzed by using Metamorph software (Molecular Devices, Downingtown, PA).

### Histology, TRAP and β-galactosidase (β-gal) Staining

Long bones were fixed in 10% formalin and then decalcified with 10% EDTA. Bones were then paraffin embedded and consecutive 4 µm sections were stained with hematoxylin and eosin (H&E) and for TRAP activity as described above. Osteoclast numbers, trabecular bone volume over total volume (BV/TV) were quantified using Metamorph software.

For β-gal staining, decalcified bones were frozen embedded and sectioned at 20 µm using a Leica Cryo-Stat. Slides were dried at room temperature and rinsed 3 times in distilled water 5 minutes to remove OCT (Sakura Finetek, Torrance, CA). X-gal was used as a substrate in a humidified chamber at 37°C incubator overnight. Alternate (adjacent) slides were used for TRAP staining. *Lac*Z+multinucleated (more than 3 nuclei/cell) TRAP positive cells were counted as mature osteoclasts.

### Zoledronic Acid Treatment

Zoledronic acid (ZA) kindly provided as the disodium salt by Pharma Novartis (Basel, Switzerland) was injected subcutaneously twice a week to myeloma bearing mice (100 µg/kg, [22]) from day 1 after tumor cell inoculation till experimental endpoint. Tumor-bearing mice without ZA treatment were used as the control group. Spleen Gr-1+/CD11b+cells from each group were sorted by magnetic beads for osteoclast culture, ZA treatment, and Western blotting.

### Western Blotting

5TGM1 cells and Gr-1+/CD11b+cells from control and ZA-treated tumor-bearing mice were lysed in RIPA buffer (components here). Lysates were separated by 10% SDS-PAGE, transferred to polyvinylidene difluoride membranes, and then blocked in TBS plus 5% nonfat milk and 0.1% TWEEN-20 at room temperature for 1 hour. Membranes were first probed with Rab6 antibody sc-310 (Santa Cruz Biotechnology, Inc. Santa Cruz, CA, 1∶200). Stripped membranes were then probed with an antibody specific for the unprenylated form of Rap1A sc-1482 (Santa Cruz Biotechnology Inc. Santa Cruz, CA). The membranes were stripped again for the detection of β-actin as a control for protein loading.

### Statistical Analysis

Data were presented as means ±SEM unless otherwise stated. Statistical significance was determined using 2-way Student’s *t*-test or 2-factor analysis of variance with replication. *P* values less than 0.05 were considered to be statistically significant.

## Results

### 5TGM1 Myeloma Cells Induce the Expansion of Gr-1+/CD11b+Cells

**Figure 1:**
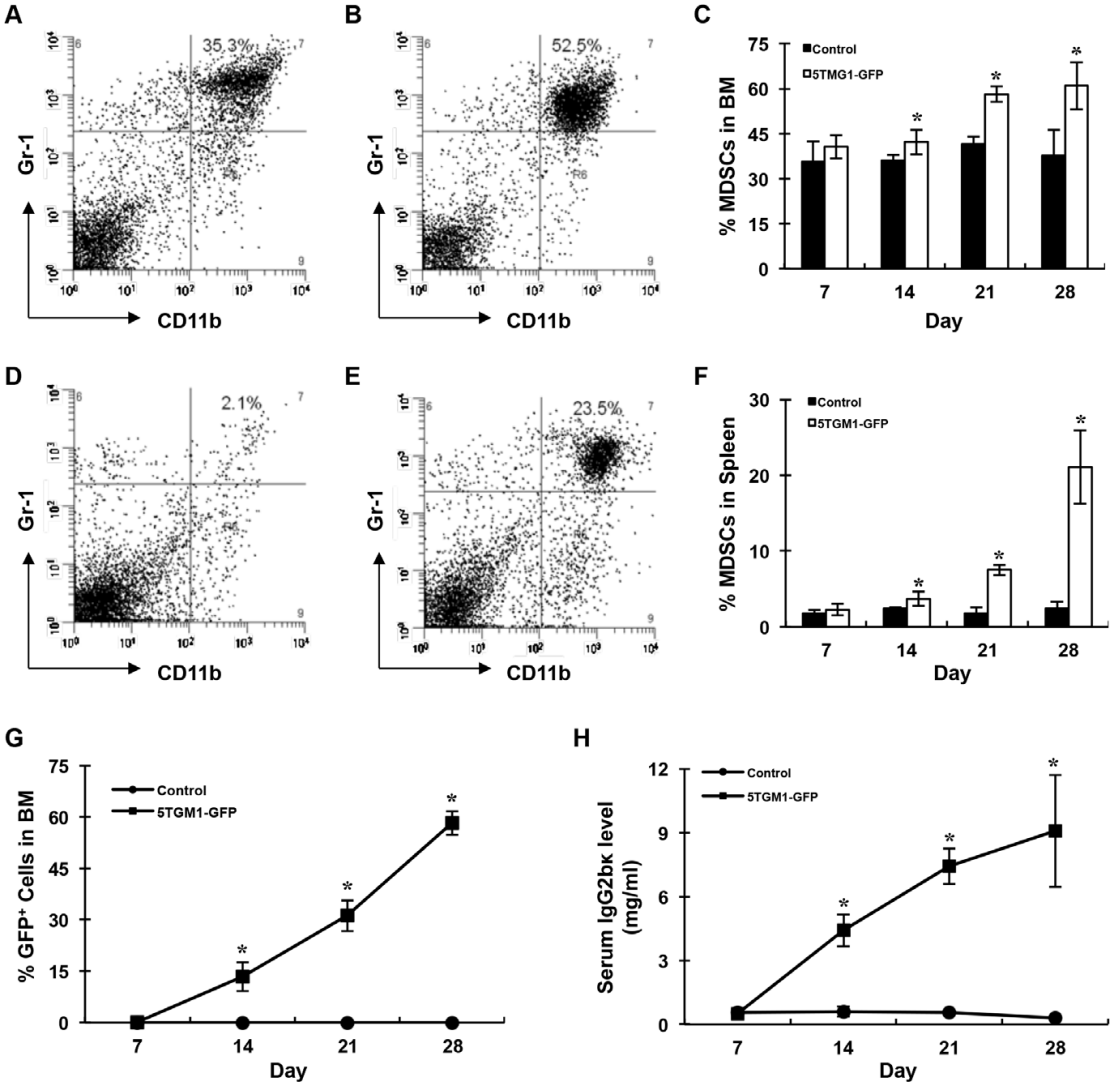
Myeloma increased Gr-1+CD11b+cells in mice. A–B, Flow cytometry analysis showing the percentage of the Gr-1+CD11b+cells in bone marrow (A) and spleen (B) of control and 5TGM1-GFP inoculated mice 28 days after tumor inoculation. **C–D,** The quantification of flow cytometry of A–B. *P<0.05, n = 6. **C∼D,** Myeloma model validation. Percentage of 5TGM1-GFP+myeloma cells (**C**) and serum IgG2bκ level (mg/ml) (**B**) in C57BL6/KaLwRij mice increased with time after 5TGM1 myeloma cell inoculation. n = 6 for tumor group; n = 4 for control group.

To validate our murine myeloma models, we first inoculated GFP-positive 5TGM1 myeloma cells into C57BL6/KaLwRij mice by tail vein injection as described previously [23]. MDSCs were isolated and assessed by flow cytometry analysis every week for 4 weeks after tumor cell inoculation. We observed a small and non-significant increase in the percentage of Gr-1+/CD11b+cells in tumor-bearing mice compared to the controls (2.24±0.75% vs. 1.78±0.41% in spleen and 37.7±8.6% vs. 35.0±0.67% in bone marrow) following 1 week of myeloma implantation. The percentage of Gr-1+/CD11b+cells in marrow, spleen, and blood in the tumor group began to increase significantly on week 2 and continued to increase with time compared with the control group. On week 4, the difference in the percentage of MDSCs between 5TGM1-bearing and control mice were 60.9±7.8% vs. 37.7±8.6% (p<0.05) in bone marrow, 21.1±4.84% vs. 2.4±0.85% (p<0.05) in spleen, and 23.1±4.27% vs. 5.4±1.1% (p<0.05) in blood, respectively (Fig. 1A, B, and data not shown). The expansion of MDSCs paralleled myeloma burden in bone and spleen as indicated by a significant increase in the number of GFP+myeloma cells in bone marrow and in the production of serum IgG2bκ, indicative of systemic tumor burden during myelomagenesis [19], in the 5TGM1-GFP bearing mice compared with control mice (Fig. 1C, D). Our data thus indicate that MM cells induce the expansion of Gr-1+/CD11b+cells in both spleen and bone marrow.

### Gr-1+/CD11b+from Myeloma-bearing Mice Differentiate into Osteoclasts in vitro

**Figure 2:**
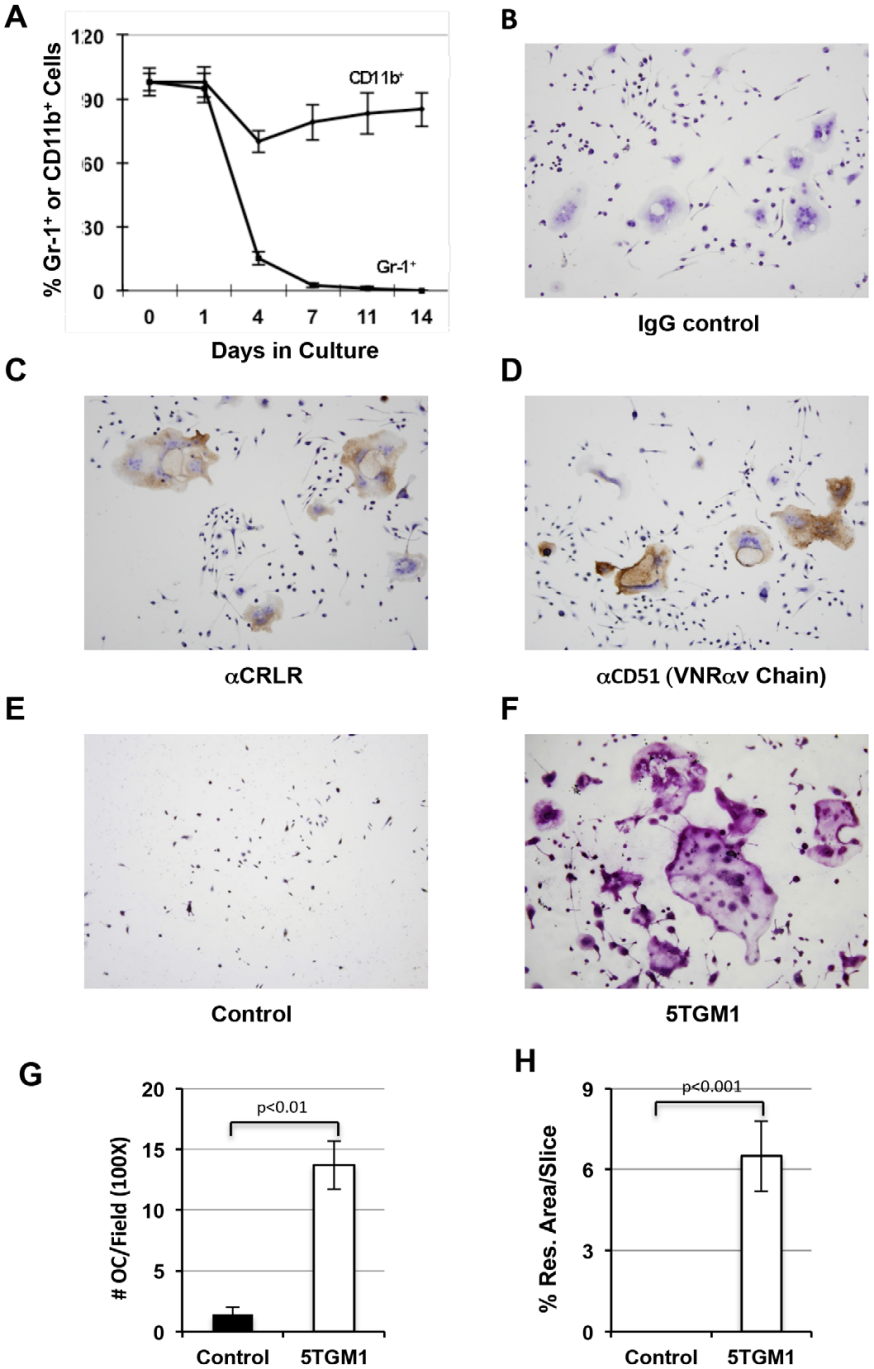
Tumor-associated MDSCs are osteoclast precursors. **A.** Gr-1 expression decreases with cultured time in MDSCs from myeloma-bearing mice. Note that FACS sorted MDSCs were double positive for both Gr-1 and CD11b surface markers (98%) at the beginning of the culture time. However, the percentage of Gr-1-expressing cells progressively dropped with cultured time. **B–D.** Immunocytochemistry using indicated antibodies. IgG, negative control (**B**); **α**CRLR, Calcitonin receptor-like receptor (**C**); αCD51 (VNRαv chain. **E**–**F**, TRAP staining of MDSC cultures of nontumor-bearing mice (control, **E**) or tumor-bearing mice (5TGM1, **F**) grown under osteoclastogenic condition for 14 days. G. Quantification of **E** and **F. H.** Dentine assays demonstrating that osteoclasts derived from MDSCs of tumor-bearing mice has greater activity to resorb bone.

To address whether the myeloma-associated Gr-1+/CD11b+cells had the potential to differentiate into functional osteoclasts that might be responsible for the increased bone lesions associated with MM, we performed in vitro osteoclast differentiation assay. Gr-1+/CD11b+cells from spleen were sorted by FACS (98% purity) and were cultured under osteoclastogenic conditions in media containing M-CSF and RANKL. Cells were then monitored by immunocytochemistry for the expression of osteoclasts surface markers. As shown in Fig. 2A, the percentage of CD11b+cells remained high throughout the course of the differentiation process. On the contrary, the Gr-1-expressing cell population declined sharply to 13% in day 4 cultures. The percentage of the Gr-1-expressing cells was only 2.5% cells in day 7 cultures. From day 11 and day 14 cultures, no Gr-1 positive cells could be detected.

Starting from culture day 7, multinucleated (≥3 nuclei) cells began to appear and their number and size continued to rise in the cultures of MDSCs from 5TGM1-bearing mice (Fig. 2B). Those CD11b+multinucleated cells reacted to antibodies against calcitonin receptor-like receptor (CRLR) and CD51 (the αv chain of the vitronectin receptor), two mature osteoclast markers [24], [25], and Tartrate-resistant acid phosphatase (TRAP+), a surface marker for mononucleated osteoclast precursors and mature osteoclasts (Fig. 2C–F). Quantitatively, there was 13.8% (±2.2%) of cells that contained 3 or more nuclei in MDSC cultures from tumor-bearing mice whereas only 1.2% (±0.7%) of multinucleated cells formed from the non-tumor-bearing controls (Fig. 2G). Accordingly, no CRLR- and CD51-positive multinucleated cells were detected in the cultures of MDSCs from non-tumor-bearing control mice (data not shown). We thus conclude that Gr-1−/CD11b+cells derived from Gr-1+/CD11b+cells of 5TGM1-bearing mice can differentiate into osteoclasts in vitro.

To determine whether the multinucleated TRAP+cells observed were able to function like osteoclasts to resorb bone, we grew the Gr-1+/CD11b+cells cells on dentine slices for 28 days. Resorption pit areas on dentine slices were measured and resorption activity was presented by the ratio of resorption area over total area. Dentine slices containing Gr-1+/CD11b+cells cells from tumor-bearing mice had 6.7% resorption pit-covered area whereas slices containing naïve Gr-1+/CD11b+cells from non-tumor-bearing control mice had no resorption pits (Fig. 2H). These data indicate that Gr-1+/CD11b+cells from myeloma-challenged mice but not from the control mice can differentiate into functional bone resorbing osteoclasts.

### Gr-1+/CD11b+Cells from Myeloma-bearing Mice Differentiate into Osteoclasts in vivo

**Figure 3:**
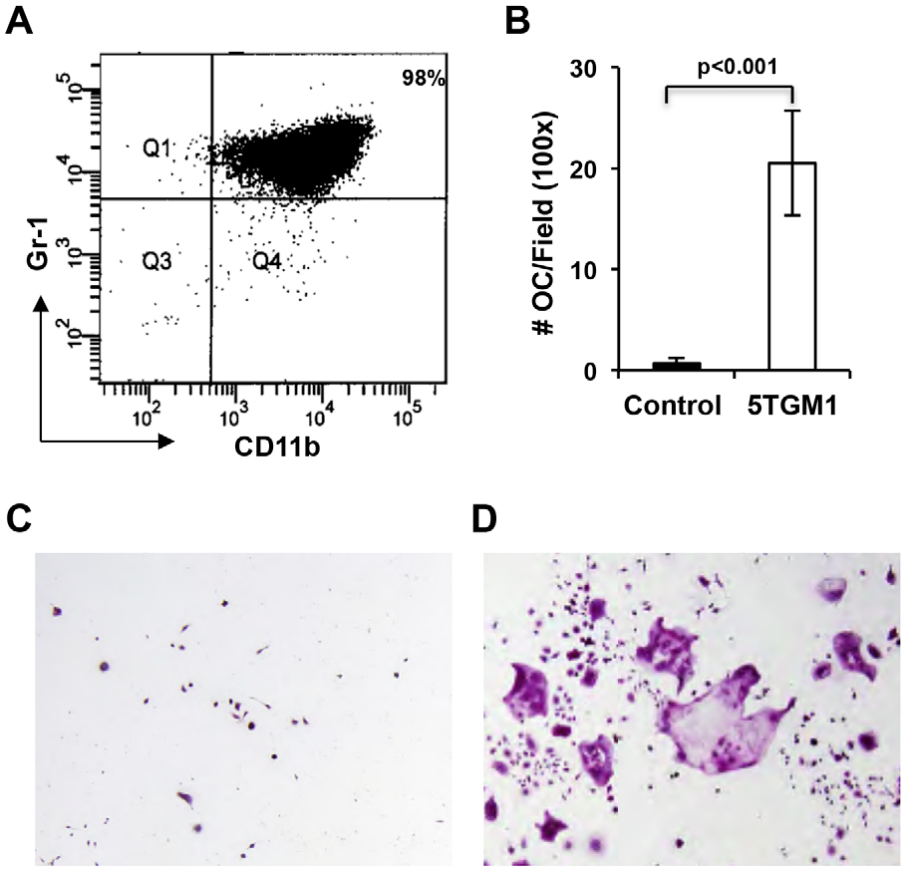
Gr-1+CD11b+cells from *Rag2* ^−/−^ myeloma-bearing mice form osteoclasts. A, Flow cytometry graph showing isolated Gr-1+CD11b+cells from 5TGM1-challenged mice. **B,** Quantification of multinucleated TRAP+cell number (# OC) of Gr-1+CD11b+cultures from nontumor-bearing control (control) or 5TMG1-challenged mice (5TGM1). Cells were cultured for 14 days under osteoclast differentiation condition. **C–D,** TRAP staining of Gr-1+CD11b+cultures from control or myeloma-bearing *Rag2* ^−/−^ mice (100×).

To determine whether the Gr-1+/CD11b+cells from myeloma-bearing mice could differentiate into osteoclasts in vivo, we established a murine myeloma mouse model that carried the *LacZ* transgene in the *Rag2* ^−/−^ immunodeficient background (*LacZ*;*Rag2* ^−/−^ mice). Since *Rag2* ^−/−^ mice develop myeloma similarly to the C57BL6/KaLwRij strain following 5TGM1-GFP inoculation [21], we reasoned that if tumor-associated MDSCs derived from *Rag2* ^−/−^ mice would also develop into osteoclasts. To confirm this, we differentiated the Gr-1+/CD11b+cells of *Rag2* ^−/−^ mice challenged with 5TGM1-GFP. Our data demonstrated that many multinucleated TRAP+cells formed in the MSDC cultures of MM-challenged *Rag2* ^−/−^ mice whereas no multinucleated TRAP+cells were observed in the MSDC cultures of naïve *Rag2* ^−/−^ mice (Fig. 3).

**Figure 4:**
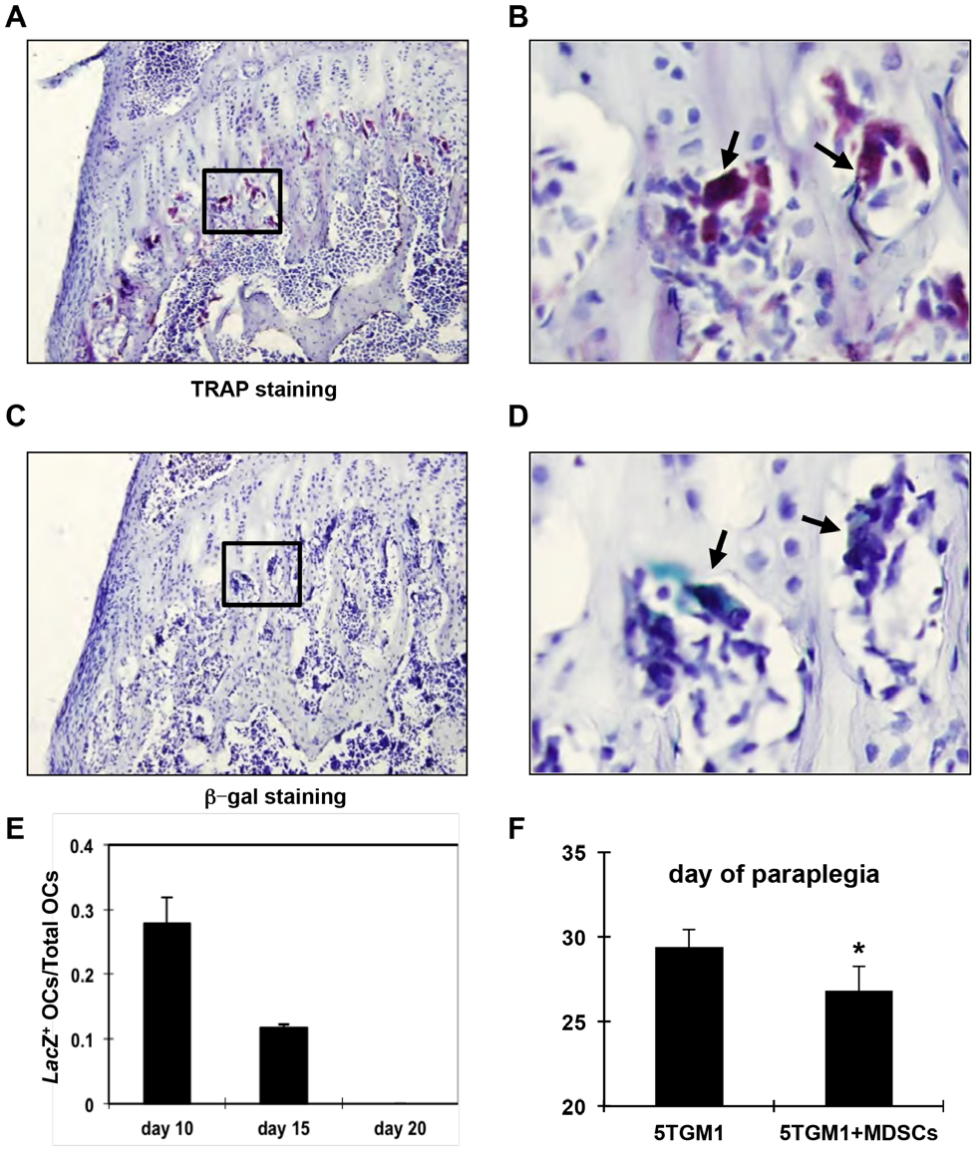
Gr-1+CD11b+cells from myeloma-bearing mice differentiate into osteoclasts in vivo. A–B. β-galactosidase staining of bone sections of tumor-bearing mice 10 days after co-inoculation of 5TGM1 cells and Gr-1+CD11b+cells of tumor-challenged *Lac*Z;*Rag2* ^−/−^ mice. Note the multinucleated *LacZ*+cells (blue) present under the growth plate in **A** (200×) and **B** (400× of the square in A). **B–C.** TRAP staining showing multinucleated osteoclasts. **C**, 200×; **D**, 400× of indicated area in **C**. **E**, The ratio of *LacZ*+osteoclasts over the total number of multinucleated TRAP+osteoclasts at indicated days after coinjection of 5TGM1 and MDSCs of myeloma-challenged *LacZTg*;*Rag2^−/−^* mice. n = 3. **F.** Tumor-associated MDSCs facilitate paraplegia in 5TGM1-induced paraplegia in mice. *p = 0.02, n = 6 for tumor group; n = 5 for control group.

Having confirmed the MDSCs behaved similarly to the ones from the C57BL6/KaLwRij mouse strain in vitro, we isolated the Gr-1+/CD11b+cells of 5TGM1-challenged *LacZ*;*Rag2* ^−/−^ mice and inoculated them into *Rag2* ^−/−^ mice. LacZ+blue osteoclasts in the mice were monitored by β-galactosidase (β-gal) and TRAP activity staining of alternate long bone frozen sections. Many TRAP+multinucleated were found in the primary spongiosa of long bones from the *Rag2* ^−/−^ mice that received both 5TGM1 and Gr-1+/CD11b+cells from 5TGM1-challenged *LacZ*;*Rag2* ^−/−^ mice (Fig. 4A, B). Few of the multinucleated TRAP+cells were also positive for β-gal activity (LacZ+), indicating that they are derived from the *LacZ*;*Rag2* ^−/−^ MDSCs (Fig. 4C, D). The percentage of LacZ+versus total TRAP^+^multinucleated cells decreased with time (27.9±3.9% and 11.8±0.4% in bones harvested at day 10 and day 15 post tumor inoculation, respectively). No LacZ+osteoclasts were found in bones harvested from these mice at 20 days post tumor induction (Fig. 3E). These data suggested that donor Gr-1+/CD11b+cells can differentiate into osteoclasts but fail to expand in host mice.

### Tumor Induced Gr-1+/CD11b+Cells Exacerbate Tumor Burden and Bone Destruction

**Figure 5:**
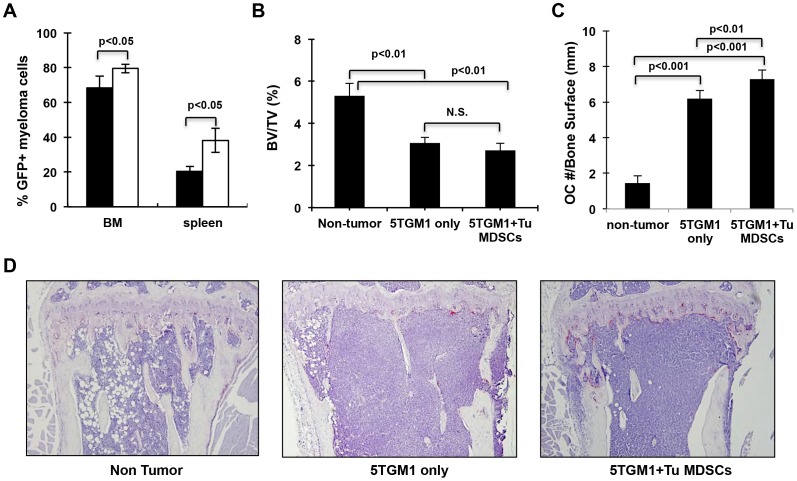
Gr-1+CD11b+cells from myeloma-bearing mice increased tumor burden and bone lesion. A. Quantification of GFP+5TGM1 cells in bone marrow and spleen of mice after co-inoculation of 5TGM1-GFP and MDSCs of myeloma-challenged or inoculation of 5TGM1 alone. **B.** Bone lesion assessed by quantification of bone volume over total volume (BV/TV) on histology of tibial sections. N.S., not significant. **C,**TRAP staining of tibial sections (100x) and **D,** quantification of OC #/bone surface showing the increased osteoclast number in 5TGM1 only and 5TGM1+Tu MDSCs groups compared with non-tumor control. Note that osteoclast numbers increased in mice coinoculated with MDSCs and 5TGM1 compared to mice inoculated with 5TGM1 alone.

Having shown that Gr-1+/CD11b+cells from myeloma-bearing mice could develop into multinucleated osteoclasts in vitro and in vivo, we asked whether expansion of Gr-1+/CD11b+cells following tumor implantation could contribute to bone resorption. To examine this, we compared the tumor burden between mice injected with 5TGM1-GFP alone and mice co-inoculated with 5TGM1-GFP and tumor-induced Gr-1+/CD11b+cells. Tumor progression was monitored by recording the number of days elapsed from the day of inoculation to the day of the appearance of paralysis in the lower limb. A small but statistically significant acceleration in the development of paraplegia was observed in mice that received both 5TGM1 and myeloma-associated Gr-1+/CD11b+cells compared to mice that received 5TMG1 alone (Fig. 4F, 29.4 (±1.02) vs. 26.8 (±1.5) days post inoculation, n = 5, p = 0.02). These data suggested that myeloma-induced Gr-1+/CD11b+cells facilitate 5TMG1 myeloma development in vivo. Supporting this, an enhanced tumor burden, as measured by the percentage of GFP+tumor cells vs. total bone marrow cells, was found in mice received both 5TGM1-GFP cells and myeloma-associated Gr-1+/CD11b+cells compared to those received 5TGM1-GFP cells alone (Fig. 5A, 79.5±2.5% vs. 68.4±6.7% in the bone marrow and 38.2±6.9% vs. 20.3±2.8% in the spleen, respectively). Furthermore, the number of osteoclasts on the bone surfaces in control mice was 1.42±0.43, which was increased to 6.2±0.45 in mice received 5TGM1-GFP cells alone, representing a 4.4-fold increase in osteoclast numbers in tumor-bearing mice. Osteoclast number was further increased 1.2-fold in mice received both 5TGM1-GFP and tumor-induced Gr-1+/CD11b+cells (Fig. 5B, compare 7.3±0.51 vs. 6.2±0.45, p<0.01). Corresponding to this enhanced increase in osteoclast numbers, a reduction in bone volume over total tissue volume (BV/TV) was observed in mice coinjected with both tumor cells and tumor-induced Gr-1+/CD11b+cells compared to mice inoculated tumor cells alone (Fig. 4C, 2.72±0.34% vs. 3.07% ±0.26%), although the difference between the two groups was not statistically significant at the late time point examined. Taken together, these results indicate that myeloma-associated MDSCs increase the number of bone marrow osteoclasts and facilitate myeloma growth, which may contribute to facilitate MM-related bone destruction in vivo.

### Zoledronic Acid Inhibits Gr-1+/CD11b+Expansion and Differentiation into Osteoclasts, Independent of its Inhibition of Prenylation

**Figure 6:**
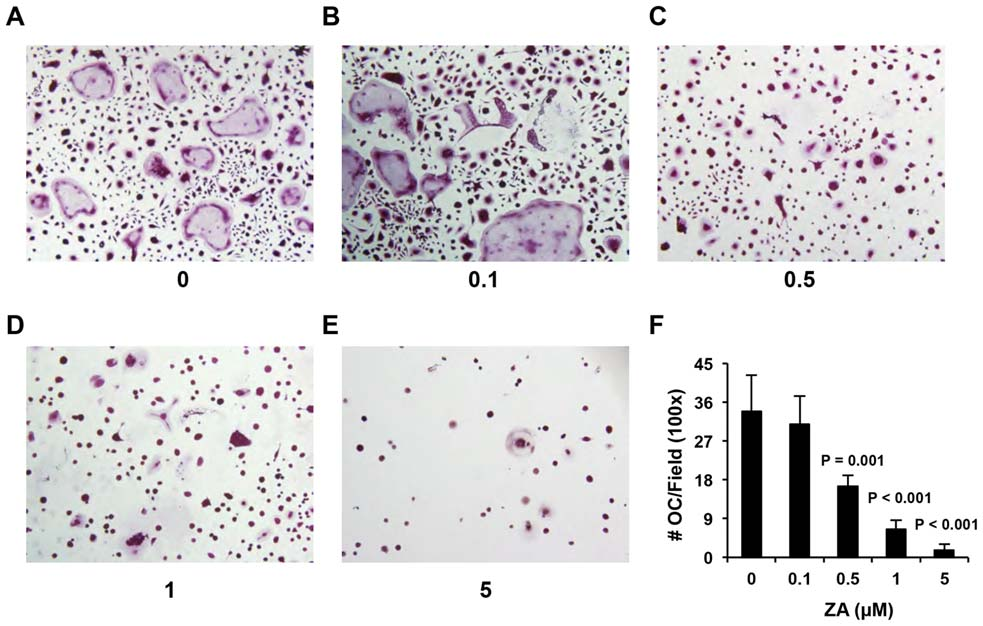
Zoledronic acid (ZA) inhibits osteoclast differentiation of myeloma-induced^+^MDSCs in vitro. A–E, Photographic images of the cell cultures of Gr-1+CD11b+cells isolated from spleen of myeloma-bearing mice. Cells were treated with ZA at indicated concentrations and TRAP staining was performed at day 14 post osteoclastogenic induction. F. Multinucleated cells were quantified as the number of multunucleated TRAP+osteoclasts per 100× field. n = 3.

Zoledronic acid (ZA) is one of the most potent nitrogen-containing bisphosphates that inhibits not only osteoclast-mediated osteolytic bone destruction but also myeloma cell growth [26], [27]. To determine whether ZA inhibited osteoclast differentiation of tumor-induced MDSCs, we cultured the Gr-1+/CD11b+cells of myeloma-challenged mice under osteoclastogenic condition in the presence of ZA at various concentrations. Consistent with our previous observations, many TRAP+multinucleated osteoclasts [33.8 (±8.5) per 100× field] were observed in the MDSC cultures. However, the number of TRAP+cells decreased to 30.8 (±6.6), 16.5 (±2.3), 6.5 (±2.1), and 1.7 (±1.4) per 100× field in the presence of 0.1, 0.5, 1, and 5 µM of ZA, respectively (Fig. 6). These data demonstrated that ZA dose-dependently inhibits osteoclast formation in the tumor-induced MDSC cultures in vitro.

**Figure 7:**
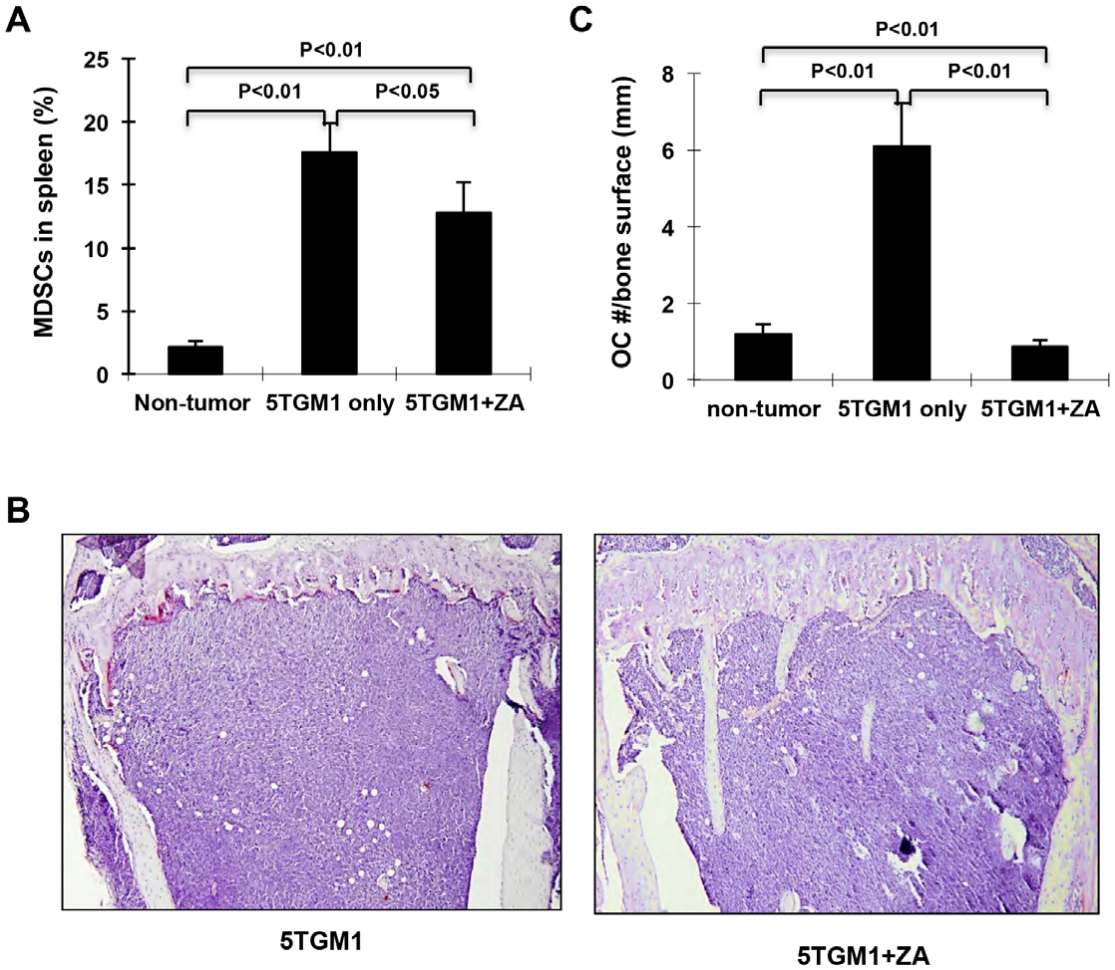
Zoledronic acid (ZA) decreases the myeloma-induced expansion of Gr-1+CD11b+cells in vivo. A. FACS analysis showing the percentage of Gr-1+CD11b+cells in spleen of control, tumor-bearing mice that were non-treated (5TGM1) and treated with ZA (5TGM1+ZA). **B,** TRAP staining of tibial bone sections from tumor-bearing mice that were non-treated (5TGM1) or treated with ZA (5TGM1+ZA). **C,** Quantification of osteoclast number over bone surface of **B.** *p<0.05, n = 5 for each group.

To examine the in vivo effect of ZA on the expansion of Gr-1+/CD11b+and osteoclast differentiation upon tumor growth, we treated non-tumor control mice and myeloma-bearing mice with ZA (0.1 mg/kg) twice a week for 4 weeks following 5TGM1 cell inoculation. As expected and consistent with data in Fig. 1, a small fraction of Gr-1+/CD11b+cells was found (2.12±0.48%) in spleen of control mice, which was increased (17.6±2.27%) in spleen of myeloma-bearing mice. However, only 12.8±2.4% of Gr-1+/CD11b+cells were observed following ZA treatment of tumor-bearing mice, indicating a 30% reduction by ZA treatment (Fig. 7A). Consistent with this, the tumor-associated increase in the number of osteoclasts per bone surface (6.1±1.12 vs. 1.2±0.13 per mm bone surface, p<0.01) in tumor-bearing mice was completely reduced to normal level by ZA treatment (0.88±0.16 vs. 1.2±0.13 per mm bone surface p<0.01, Fig. 7B, C). Consistent with the in vitro data in Fig. 6, ZA strongly inhibited of osteoclastogenesis of the Gr-1+/CD11b+cells of ZA-treated and non-treated control mice, as shown by a 40% reduction in the number of TRAP+cells formed in the Gr-1^+^/CD11b^+^cultures of control vs. ZA-treated tumor-bearing mice (42.4±4.0 vs. 25.6±3.5 osteoclasts per 100× field, p<0.01, Fig. 7).

To further understand the molecular basis by which ZA inhibited the differentiation of tumor-induced MDSCs into osteoclasts, we analyzed the prenylation of two small GTPase, Rap1A and Rab6, which are important intracellular signaling proteins that are activated by prenylation. ZA has been shown to inhibit the prenylation of Rap1A and Rab6, which leads to impairment in osteoclast maturation, survival, and recruitment of osteoclasts to bone resorption sites [28]–[32]. Since our study demonstrated that ZA treatment of human myeloma cells increases the abundance of the non-prenylated form of Rap1A and decreases the prenylated form of Rab6 [27], thus we treated 5TGM1 mouse myeloma cells with increased concentration of ZA as a positive control. Consistent with our previous observation in human myeloma cells, ZA increased the amount of non-prenylated Rap1A dose-dependently and decreased prenylated Rab6 at high concentration. However, we did not detect any difference in the prenylation of Rap1A and Rab6 in the Gr-1+/CD11b+cells from spleen of either non-treated or ZA-treated tumor-bearing mice (Fig. 6C). Therefore, ZA may inhibit the differentiation of Gr-1+/CD11b+cells from tumor-bearing mice by a mechanism independent of inhibition of protein prenylation.

## Discussion

**Figure 8:**
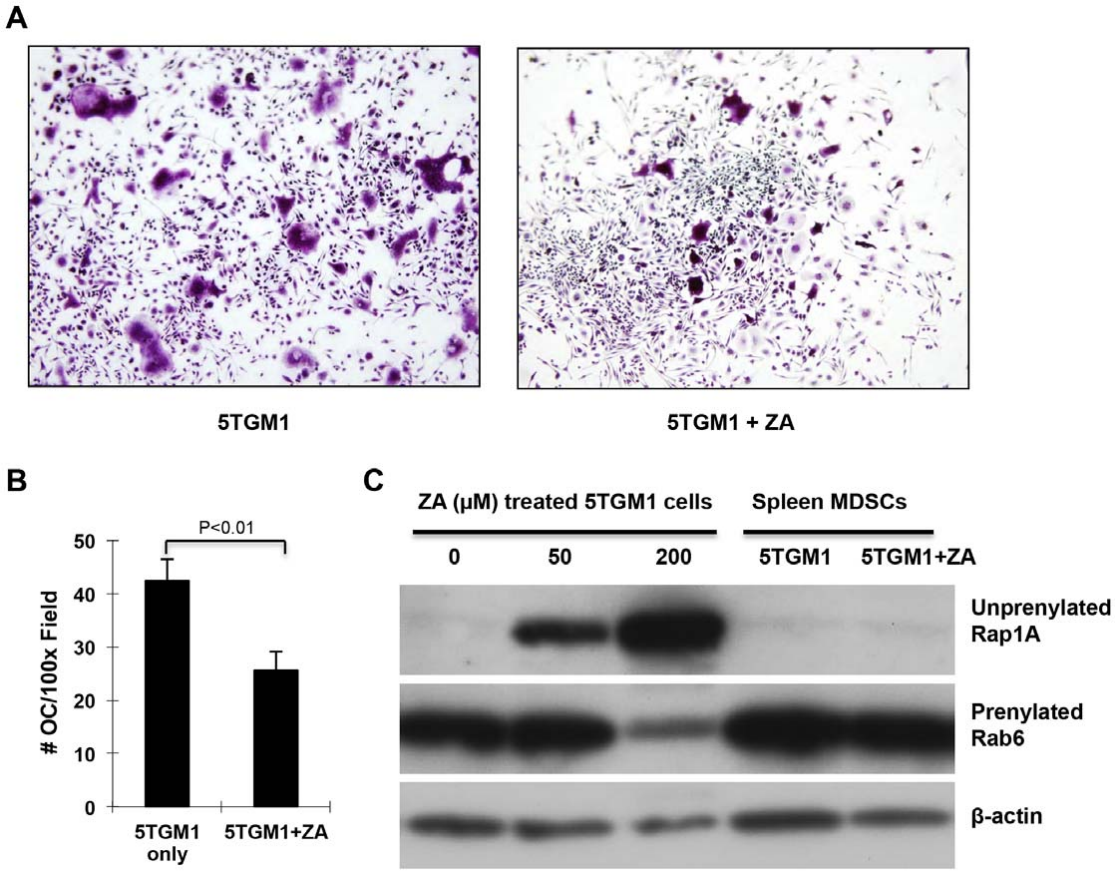
ZA inhibits osteoclast differentiation of Gr-1+CD11b+cells via a prenylation-independent manner. A. TRAP staining of Gr-1+CD11b+cultures of tumor-bearing mice nontreated (5TGM1) or treated with ZA under osteoclast differentiation medium for 14 days (100×). **B.** Quantification of osteoclast number of **A.** **C.** Western blot showing that in vivo treatment of ZA does not affect prenylation of Rap1A and Rab6 in MDSCs of tumor-bearing mice. Note that prenylation of Rap1A and Rab6 were inhibited by ZA treatment in 5TGM1 cells in vitro.

Our understanding of the mechanisms by which tumor-associated MDSCs promote cancer growth has improved over the last decade. In this study, we provide evidence that MDSCs induced by multiple myeloma (MM) can differentiate into osteoclasts in vitro and in vivo (Figs. 1, 2, 3, 4), and that myeloma-associated MDSCs facilitate myeloma development, increase tumor burden and bone resorption (Fig. 5). We also find that bisphosphonates reduce the potency of MDSCs to differentiate into osteoclasts (Figs. 6, 7) and interestingly this function is independent of its ability to inhibit prenylation of small GTPases (Fig. 8).

The *LacZ* transgene-containing *Rag2* ^−/−^ mouse model created in this study has provided a useful tool to trace the in vivo differentiation of transplanted MDSCs. By coinjecting 5TGM1 and *LacZ* ^+^tumor-associated MDSCs in *Rag2* ^−/−^ mice, we were able to detect LacZ and TRAP-positive multinucleated osteoclasts in host bones (Fig. 4), providing evidence that tumor-associated MDSCs are precursors of osteoclasts. In addition, our results indicate that tumor-associated MDSCs do not have the capacity to proliferate in recipient mice, as shown by the low proportion of LacZ+osteoclasts in host bones and decline over time (Fig. 4E). Zoledronate treatment of myeloma-bearing mice resulted in a decrease in the expansion of tumor-induced MDSCs, and a reduction in their ability to form osteoclasts compared to vehicle treated mice (Figs. 6, 7, 8). No evidence of inhibition of protein prenylation was detected in MDSCs isolated from myeloma-bearing mice treated with zoledronate, suggesting that this effect of bisphosphonates on MDSCs is independent of the characteristic inhibition of protein prenylation in mature osteoclasts.

MDSC populations from non-tumor host contain mostly committed neutrophils [8], which have lost the capacity to differentiate into other cell types. Tumor-associated MDSCs can differentiate into immature dendritic cells that fail to efficiently present antigen to T-lymphocytes [6], [33], which suppresses immunity. Furthermore, they not only secrete cytokines, such as VEGF, MMP9, and TGFβ, to support angiogenesis in tumors, but also serve directly as precursors to form tumor endothelium in vivo, which facilitates tumor growth and tumor invasion [8]. Complementing these previous studies, we show that tumor associated MDSCs promote tumor burden in both bone marrow and spleen of host (Fig. 4). These studies thus indicate that tumor-associated MDSCs have greater plasticity and broader potential than naïve MDSCs, which are known to suppress anti-tumor immunity in the host. The contribution of tumor-induced MDSC populations of myeloid cells to the increase in tumor burden and osteolysis in MM was unclear. Our data suggest that tumor-associated MDSCs are not the same as naïve or non disease-associated MDSCs since the latter cannot be induced to differentiate into osteoclasts nor can they resorb bone (Figs. 2, 3). This result suggests that tumor-associated MDSCs and normal MDSCs may have different molecular signatures, albeit they both express Gr-1 and CD11b. Liu et al. [34] recently found that naïve MDSCs express higher level of miR-223 than tumor-associated MDSCs. Interestingly, miR-223 expression is downregulated by tumor-associated factors and is a strong suppressor of Gr-1+/CD11b+cell differentiation. Kim et al. [35] recently reported that MDSCs obtained at a late time point after tumor injection express higher levels of tumor function-enhancing genes and lower levels of immune response-related genes than MDSCs obtained at an early time point. The molecular mechanisms governing the differential potentials of naïve and tumor-associated MDSCs to differentiate into osteoblasts are largely unknown and worthy of investigation.

Although this study focuses on MM-associated MDSCs, an increase in the proportion of granulocytic MDSCs, defined as Gr-1+/CD11b+, was demonstrated in the long bones of a syngeneic mouse model of breast cancer bone metastasis [36]. These data thus suggest that both blood and solid cancers may promote MDSC expansion and that MDSCs may contribute to the formation of osteolytic lesions in these cancers. Neovascularization plays an important role in the pathogenesis of MM and myeloma plasma cells in majority of MM patients express VEGF. Thalidomide has been used as a common therapy for MM patients because of its antiangiogenic activity [37], [38]. Since tumor-associated MDSCs can stimulate neovascularization by forming tumor endothelium in other cancer models [8], it is possible that myeloma-associated MDSCs can form tumor endothelium during myelomagenesis. Complementing the previous demonstration that tumor-associated MDSCs could form tumor endothelium [8], our current study shows these cells are critically important to the skeletal complications associated with MM. Methods of depleting the MDSC population may thus be used to inhibit osteoclastogenesis and angiogenesis to reduce tumor burden and bone lesion in cancer patients.
